# COVID-19 Infection and Professional Commitment Among Clinical Nurses: A Cross-Sectional Study of Work Stress and Social Support in Eastern Taiwan

**DOI:** 10.3390/nursrep16090309

**Published:** 2026-08-30

**Authors:** Zhe-Ning Yu, Tai-Chu Peng

**Affiliations:** 1 Department of Nursing, Hualien Tzu Chi Hospital, Buddhist Tzu Chi Medical Foundation, Hualien 970, Taiwan; 2 Department of Nursing, College of Medicine, Tzu Chi University, Hualien 970, Taiwan

**Keywords:** COVID-19, nurses, work stress, social support, professional commitment, cross-sectional study

## Abstract

**Background/Objectives**: Nurses in Taiwan faced markedly increased nurse-to-patient ratios and a high risk of occupational infection during the COVID-19 pandemic, generating considerable psychological and social strain. Professional commitment—reflecting nurses’ identification with, investment in, and intention to remain in the profession—is important for workforce retention and care quality, yet little is known about how contracting COVID-19 affects it. This study examined whether COVID-19 infection and the presence of long COVID symptoms were associated with professional commitment among clinical nurses, and identified sociodemographic, work-stress, and social-support correlates of commitment. **Methods**: A cross-sectional survey was conducted among registered nurses at a medical center in eastern Taiwan between 21 September 2023 and 28 February 2024. An anonymous electronic questionnaire assessed sociodemographic characteristics, COVID-19 infection/long-COVID status, work stress (Stress Scale for Healthcare Workers Caring for Patients with High-Risk Infectious Diseases), social support (Social Support Scale for clinical nurses), and professional commitment (Hospital Nurses’ Job Satisfaction and Professional Commitment Scale). Data from 364 valid responses (response rate 39.8% of the 914 nurses employed at the study hospital) were analyzed using independent-sample *t*-tests, Pearson correlation, one-way ANOVA, and multiple linear regression. **Results**: Professional commitment did not differ significantly by COVID-19 infection status (t = 0.50, *p* = 0.619) or by presence of long COVID symptoms (t = 0.85, *p* = 0.397). Social support was positively correlated with professional commitment (r = 0.261, *p* < 0.001), as was work stress, although the latter association was weak (r = 0.116, *p* = 0.027). Age, job rank, years of service, and education level were significantly associated with commitment (all *p* < 0.05). Multiple regression indicated that higher social support (B = 0.206, *p* < 0.001), greater work stress (B = 0.079, *p* = 0.006), and older age were independently associated with higher professional commitment (R^2^ = 0.297). **Conclusions**: In this cross-sectional sample, COVID-19 infection and long COVID symptoms did not appear to be associated with nurses’ professional commitment. Social support, age, and work stress emerged as the correlates most consistently related to commitment, with social support showing the largest association. Given the observational design and the retrospective measurement of work stress and social support, these findings should be regarded as preliminary and hypothesis-generating rather than conclusive. They tentatively indicate that workplace social support may merit further investigation as a potential avenue for sustaining nurses’ professional commitment during infectious disease outbreaks.

## 1. Introduction

In December 2019, a cluster of pneumonia cases of unknown origin emerged in Wuhan, China, most linked to exposure to a seafood market [1]. The causative pathogen was subsequently identified as a novel coronavirus and named severe acute respiratory syndrome coronavirus 2 (SARS-CoV-2); the disease it causes was designated coronavirus disease 2019 (COVID-19) [1]. On 11 March 2020, the World Health Organization (WHO) characterized COVID-19 as a pandemic, and healthcare systems worldwide—including Taiwan’s—came under unprecedented strain [1,2].

Between 22 January 2020 and 24 December 2022, more than 651 million COVID-19 cases and 6.67 million deaths were recorded globally [1]; in Taiwan, cumulative confirmed cases exceeded 8.68 million, with 15,075 deaths (case-fatality rate 0.17%) [3]. Healthcare workers (HCWs) globally experienced high occupational infection risk; a systematic review across 980,000 HCWs estimated a PCR-positivity rate of 6.1% to 8.1% across two successive six-month periods in 2020 and 2021 [2]. The COVID-19 pandemic substantially increased nursing workloads and contributed to heightened turnover intention among nurses across multiple healthcare systems [4]. As the HCWs with the most direct and prolonged patient contact, nurses bore both the highest occupational infection risk and primary responsibility for maintaining infection-control integrity; breaches in protective measures therefore threatened not only their own safety but also unit staffing levels, compounding psychological and social stress [2,5].

Survey evidence from the pandemic period documents marked deterioration in nurses’ mental health globally. A systematic review and meta-analysis reported pooled prevalence rates of anxiety (32%), stress (40.6%), depression (32%), post-traumatic stress disorder (18.6%), and insomnia (38.3%) among nurses during the COVID-19 pandemic [5]. Principal stressors included personal protective equipment burden, fear of infecting family members, heavy patient-care loads, and occupational uncertainty [5]. These findings highlight the disproportionate psychological burden borne by nurses as the frontline healthcare worker group with the most sustained direct patient exposure.

Professional commitment refers to an attitude that binds an individual physically, psychologically, and emotionally to their occupation, encompassing belief in the profession’s goals and values, willingness to work toward those values, and a desire to maintain membership within it [6]. In nursing, professional commitment reflects nurses’ loyalty to, and investment in, their professional role, together with their affirmation of its value [7], and has been linked to nurses’ intention to remain in the profession and, ultimately, to the continuity and quality of patient care [7,8]. Evidence gathered during the pandemic suggests that professional commitment among nursing staff develops through an ongoing process of social interaction and self-reflection, and that negative emotional reactions (worry, fear, helplessness), insufficient recognition of the nursing role, heavy workloads, staff shortages, and hazardous working conditions may all erode this commitment [9].

Social support—the perception of being cared for, esteemed, and able to draw on assistance from family, colleagues, and supervisors—has emerged as one of the most consistent protective correlates of nurses’ work-related attitudes. A recent systematic review and meta-analysis of 38 studies involving 63,989 clinical nurses reported a significant medium-sized negative association between social support and turnover intention [10], an outcome closely linked to professional commitment [7]. In Taiwanese nursing samples specifically, social support and perceived organizational support have been positively associated with professional commitment both before the pandemic [11] and among newly graduated nurses, for whom professional commitment mediated the association between social support and intention to stay [12]. During the COVID-19 pandemic, social support was further identified as a factor sustaining nurses’ psychological functioning under acute occupational strain [4,5]. Taken together, this evidence positions social support as a central job resource whose relationship with professional commitment warranted direct examination in a pandemic-exposed nursing sample.

Work-related stress, conversely, is among the most frequently documented threats to nurses’ professional attitudes. Excessive workload, infection risk, and protective-equipment burden during the COVID-19 pandemic were associated with elevated psychological distress and turnover intention among nurses internationally [4,5], and pre-pandemic Taiwanese evidence likewise linked higher work stress to lower professional commitment among public health nurses [11]. Sociodemographic characteristics–including age, education, job rank, and years of service–have also been repeatedly associated with professional commitment in nursing samples [9,11,13], plausibly because accumulated clinical experience, career investment, and role seniority strengthen occupational identification over time [13]. These variables were therefore included both as potential correlates in their own right and as covariates when estimating the associations of work stress and social support with professional commitment.

### Theoretical Framework

This study is grounded in the Job Demands–Resources (JD-R) model [14,15], which proposes that the characteristics of any occupation can be organized into two broad categories: job demands, defined as the physical, psychological, social, or organizational aspects of a job that require sustained effort and are therefore associated with certain physiological or psychological costs, and job resources, defined as the aspects of a job that help employees achieve their work goals, reduce job demands, or stimulate personal growth. The JD-R model further proposes two underlying processes: a health-impairment process, in which excessive job demands deplete employees’ energy and are associated with strain-related outcomes, and a motivational process, in which job resources foster engagement and positive work-related outcomes, including organizational and professional commitment [14,15]. Within this framework, COVID-19 infection and the associated work stress experienced by nurses during the pandemic can be conceptualized as job demands, whereas social support from family, colleagues, and supervisors can be conceptualized as a job resource. The JD-R model would accordingly predict that job demands are associated with strain and, potentially, reduced professional commitment through the health-impairment process, whereas job resources are associated with greater professional commitment through the motivational process. This theoretical framework guided the formulation of the present study’s hypotheses.

Despite this evidence, little research has directly examined whether nurses who personally contracted COVID-19 pneumonia experienced a measurable change in their professional commitment, or how this experience interacts with work stress and social support—two correlates of professional commitment that are well established in the broader nursing literature [9,11,12,13]. This gap was motivated by the lead author’s own experience of contracting COVID-19 while working as a frontline nurse, including concern for infected colleagues and family and uncertainty about her professional role—an experience that prompted the present inquiry into whether such experiences systematically affect nurses’ professional commitment more broadly. Clarifying these relationships could inform institutional strategies to sustain nursing workforce retention and care quality during future infectious disease outbreaks.

Accordingly, and guided by the JD-R model, this study aimed to examine (1) whether COVID-19 infection and the presence of long COVID symptoms—conceptualized as job demands—were associated with professional commitment among clinical nurses, and (2) which sociodemographic factors, together with work stress (a further job demand) and social support (a job resource), were associated with professional commitment among nurses who had lived through the COVID-19 pandemic. All hypotheses were directional and were specified a priori, consistent with the one-tailed parameters used in the power analysis reported in Section 2.2. Consistent with the health-impairment process of the JD-R model, in which sustained job demands deplete personal energy and erode work-related attitudes:

**H1.**  *Nurses who had contracted COVID-19, and those reporting long COVID symptoms, would report lower professional commitment than their unaffected counterparts, because infection constitutes a direct and personally experienced occupational demand.*

**H2a.**  *Higher work stress—a further job demand—would be associated with lower professional commitment.*

Consistent with the motivational process of the JD-R model, in which job resources support goal attainment and sustain occupational engagement:

**H2b.**  *Higher social support would be associated with higher professional commitment.*

Because sociodemographic characteristics index accumulated career investment and role seniority rather than demands or resources as such:

**H2c.**  *Older age, longer tenure, and more senior job rank would be associated with higher professional commitment.*

## 2. Materials and Methods

### 2.1. Study Design and Setting

This study used a cross-sectional, structured-questionnaire design, reported in accordance with the Strengthening the Reporting of Observational Studies in Epidemiology (STROBE) guidelines for cross-sectional studies, to examine the influence of sociodemographic characteristics, work stress, and social support on professional commitment among clinical nurses who had lived through the COVID-19 pandemic, with particular attention to whether nurses who had personally contracted COVID-19 differed from uninfected colleagues. Data were collected at a medical center in eastern Taiwan between 21 September 2023 and 28 February 2024. The study protocol was originally planned to commence on 1 September 2023; however, the IRB review process extended through 21 September 2023, and data collection therefore commenced on the date of IRB approval.

### 2.2. Participants and Sampling

The target hospital employed 914 nurses at the time of the study. The required sample size was estimated using G*Power 3.1 (*t*-test, linear bivariate regression, one group, one-tailed, α = 0.05, power = 0.90), yielding a minimum requirement of 374 participants. Allowing for an anticipated 10% rate of invalid responses, the recruitment target was set at 420. Based on the prior literature reporting infection rates of approximately 30% among healthcare workers, we conservatively estimated that 25% of respondents (*n* ≈ 105–126) would have contracted COVID-19. To maximize the likelihood of direct patient-care exposure, the survey was distributed preferentially to medical, surgical, and COVID-19-dedicated wards, as well as to emergency departments and medical, surgical, and dedicated intensive care units.

Inclusion criteria were as follows: full-time, licensed registered nurses employed at the study hospital, aged ≥18 years, with ≥3 months of clinical tenure, who provided consent to complete the questionnaire. Nurses not meeting these criteria were excluded. Of 371 questionnaires returned, 364 were valid after excluding ineligible or incompletely answered responses. Relative to the 914 nurses employed at the study hospital, this corresponds to a survey response rate of 39.8% (364/914); the valid sample also exceeded the minimum required sample size of 374 estimated a priori. Because participation was voluntary and fewer than half of the eligible nursing workforce responded, the possibility of self-selection bias—for example, if nurses with stronger professional commitment or more salient pandemic experiences were more inclined to participate—cannot be excluded and is addressed in Section 4.7.

### 2.3. Measures

Table 1 summarizes the three standardized instruments used in this study, together with the self-developed sociodemographic form.

#### 2.3.1. Sociodemographic and Clinical Variables

A self-developed form collected age, sex, marital status, number of children, education level, clinical unit, years of service, job rank, COVID-19 infection status, time since infection, and presence of long COVID symptoms. Job rank was recorded using the study hospital’s internal clinical-ladder and nurse-practitioner categories: N/N1 (novice to advanced-beginner staff nurse), N2, N3, and N4 (progressively more senior clinical-ladder levels), HN/AHN (head nurse/assistant head nurse), and NP (nurse practitioner); these categories denote ascending clinical seniority from N/N1 to HN/AHN and were used as entered for the group comparisons reported in Section 3. Clinical unit was recorded as general ward, outpatient department, emergency department, critical care unit, or other.

#### 2.3.2. Work Stress

Work stress was measured with the Stress Scale for Healthcare Workers Caring for Patients with High-Risk Infectious Diseases, originally developed and validated during the SARS outbreak by Chuang and Luo [16]. Higher scores indicate greater work-related stress.

#### 2.3.3. Social Support

Social support was measured with a scale developed by Ni for clinical nurses in Taiwan [17], assessing perceived support from family/friends, colleagues, and supervisors across seven dimensions. Higher scores indicate greater perceived social support. The scale source [17] is an unpublished master’s thesis (National Taiwan University, 1998); no peer-reviewed publication reporting its psychometric validation could be identified, and the primary evidence for its performance in this study is therefore the internal consistency reported in Section 3.1 and Table 2.

#### 2.3.4. Professional Commitment

Professional commitment, the primary outcome, was assessed using the Hospital Nurses’ Job Satisfaction and Professional Commitment Scale developed by Lin and colleagues [18], covering professional identity, investment, and retention. Higher scores indicate stronger professional commitment.

### 2.4. Data Collection Procedure

The electronic questionnaire was pilot-tested with 15 nurses and revised on the basis of their feedback before formal data collection began. After review and approval by the hospital’s Department of Nursing, recruitment information was distributed through the institutional e-mail system to nursing staff. Interested nurses reviewed the study information sheet, provided electronic informed consent, and accessed the questionnaire via a QR code embedded in the recruitment e-mail. Because data collection took place after the acute phase of the pandemic had subsided, the questionnaire instructions directed participants to answer the work stress and social support items with reference to their experience during the COVID-19 pandemic period, rather than to their circumstances at the time of completion; the professional commitment items were answered with reference to participants’ current attitudes toward the profession. Responses to the work stress and social support scales are therefore retrospective in nature, and the implications of this for recall bias are considered in Section 4.7.

### 2.5. Ethical Considerations

This study was approved by the Research Ethics Committee of Hualien Tzu Chi Hospital, Buddhist Tzu Chi Medical Foundation (REC No. IRB112-163-B; approved 21 September 2023; approval period 21 September 2023 to 30 April 2024). The study was non-invasive and observational. All participants were informed of the study purpose, procedures, and the scope of data use before providing electronic informed consent. Participant data were coded to protect confidentiality, with access restricted to the investigators, the academic supervisor, and the ethics committee; data were used solely for academic research. Participants were informed of their right to withdraw at any time without penalty.

### 2.6. Statistical Analysis

Data were analyzed using IBM SPSS Statistics, version 25.0 (IBM Corp., Armonk, NY, USA). Descriptive statistics (frequencies, percentages, means, standard deviations) characterized the sample. Internal consistency of each scale in this sample was assessed with Cronbach’s alpha. Independent-sample *t*-tests compared scale scores by COVID-19 infection status and by long-COVID symptom status. Pearson correlation coefficients examined associations among the three scales. One-way analysis of variance (ANOVA) with least-significant-difference (LSD) post hoc tests examined associations between sociodemographic variables and professional commitment. For the inferential analyses, age was collapsed from the seven descriptive categories shown in Table 3 into three groups (18–30, 31–40, and ≥41 years) and years of service into four groups (0–2, 3–5, 6–10, and ≥10 years) in order to obtain adequate numbers of participants in each category for group comparison; the descriptive categories reported in Table 3 were retained unchanged for sample description. Least-significant-difference post hoc comparisons were used because this part of the analysis was exploratory in nature, aiming to identify candidate sociodemographic correlates of professional commitment for confirmation in future research rather than to test a small number of pre-specified pairwise hypotheses; we acknowledge that LSD does not control the family-wise error rate, and the corresponding post hoc findings (Table 7) should be interpreted as hypothesis-generating rather than confirmatory. Multiple linear regression was used to identify independent correlates of professional commitment, entering work stress, social support, and sociodemographic variables as predictors. Distributional assumptions for the parametric tests were not formally assessed; given the sample size (*N* = 364), the analyses relied on the robustness of *t*-tests, ANOVA, and ordinary least-squares regression to moderate departures from normality in large samples, and this reliance is acknowledged as a limitation in Section 4. Because all study variables were obtained from a single self-report instrument administered at one time point, Harman’s single-factor test was conducted to assess the potential influence of common method variance: all 72 items from the three scales were entered into an unrotated principal components analysis with the number of extracted factors fixed at one. To address the possibility that nurses recruited after the pandemic peak might have diluted the associations of interest, a pre-specified sensitivity analysis repeated the *t*-tests, correlation analysis, and multiple regression in the subsample of nurses with ≥3 years of tenure. A *p*-value of less than 0.05 was considered statistically significant.

## 3. Results

A total of 364 valid responses were analyzed, as described in Section 2.2.

### 3.1. Reliability of the Instruments and Common Method Variance

Cronbach’s alpha for each instrument, as observed in this sample, is shown in Table 2 alongside the values reported for the original scales. All three instruments showed good internal consistency in this sample, equal to or exceeding the reliability reported for their original versions.

Harman’s single-factor test indicated that common method variance was unlikely to bias the findings. When all 72 items from the three instruments were entered into an unrotated principal components analysis, the analysis yielded 12 components with eigenvalues greater than 1, and the first (largest) component accounted for 21.85% of the total variance—substantially below the commonly applied 50% threshold. This pattern indicates that no single factor dominated the covariance among the measured variables.

### 3.2. Sample Characteristics

Table 3 presents the sociodemographic characteristics of the 364 participants. Most participants were women (90.9%), aged 18–30 years (54.9%), held a bachelor’s degree (75.0%), and worked on general wards (45.6%). The majority (86.8%, *n* = 316) reported having contracted COVID-19; among these, 45.9% (*n* = 145) reported experiencing long COVID symptoms.

### 3.3. Descriptive Statistics of Scale Scores

Table 4 presents descriptive statistics for the total and subscale scores of the three instruments.

### 3.4. COVID-19 Infection Status, Long COVID Symptoms, and Scale Scores

Independent-sample *t*-tests showed no significant differences in work stress, social support, or professional commitment scores between nurses who had and had not contracted COVID-19, or between infected nurses with and without long COVID symptoms (Table 5).

### 3.5. Correlations Among the Three Scales

Pearson correlation analysis (Table 6) showed that work stress was not significantly correlated with social support (r = −0.006, *p* = 0.903). Both work stress (r = 0.116, *p* = 0.027) and social support (r = 0.261, *p* < 0.001) were significantly and positively correlated with professional commitment, although the correlation between work stress and professional commitment was weak in magnitude despite reaching statistical significance.

### 3.6. Sociodemographic Correlates of Professional Commitment

One-way ANOVA with LSD post hoc comparisons (Table 7) showed that age, job rank, years of service, and education level were significantly associated with professional commitment scores (all *p* < 0.05), whereas clinical unit was not (*p* = 0.323). Commitment scores were higher among nurses aged 31–40 and ≥41 years than among those aged 18–30 years; higher among senior ranks (HN/AHN) than among N/N1, N2, and N3; higher among nurses with ≥10 years of service than among those with fewer years of service; and higher among nurses with a graduate degree than among those with a bachelor’s or associate/college degree.

### 3.7. Multiple Regression Analysis of Factors Associated with Professional Commitment

A multiple regression model (general linear model, Type III sum of squares) entering work stress and social support as covariates together with the sociodemographic variables listed in Table 8 was significant overall (F(22, 341) = 6.550, *p* < 0.001; R^2^ = 0.297, adjusted R^2^ = 0.252). The “long COVID symptoms” variable was excluded from this model because it was collinear with COVID-19 infection status (by definition, every uninfected participant necessarily had no long COVID symptoms), which made the original model rank-deficient; after this variable was removed, COVID-19 infection status was included with proper degrees of freedom and was not significantly associated with professional commitment. Of the remaining predictors, only work stress, social support, and age contributed significant unique variance (Table 8). In the final model (Table 9), higher social support (B = 0.206, *p* < 0.001), greater work stress (B = 0.079, *p* = 0.006), and older age were each independently associated with higher professional commitment scores.

### 3.8. Sensitivity Analysis Restricted to Nurses in Practice During the Pandemic Peak

Because the eligibility criterion of ≥3 months of clinical tenure permitted the inclusion of nurses recruited after the peak of the pandemic, a sensitivity analysis was conducted restricted to nurses with ≥3 years of tenure (*n* = 286), all of whom were in practice before 2022 and were therefore employed during the period of peak COVID-19 caseload in Taiwan. The independent-sample *t*-tests, correlation analysis, and multiple regression were repeated in this restricted sample.

The pattern of findings was unchanged. Professional commitment again did not differ significantly by COVID-19 infection status (t = 1.299, df = 284, *p* = 0.195) or by the presence of long COVID symptoms (t = 0.488, df = 249, *p* = 0.626). Work stress (r = 0.140, *p* = 0.018) and social support (r = 0.290, *p* < 0.001) remained positively correlated with professional commitment, with correlation coefficients marginally larger than those obtained in the full sample.

In the restricted-sample regression (*n* = 222 after listwise deletion of cases with missing values on the model covariates; F(19, 202) = 3.970, *p* < 0.001; R^2^ = 0.272, adjusted R^2^ = 0.203), social support (F(1, 202) = 19.360, *p* < 0.001; B = 0.175), work stress (F(1, 202) = 7.787, *p* = 0.006; B = 0.108), and age (F(2, 202) = 7.828, *p* = 0.001) again emerged as the only significant predictors, with all coefficients in the same direction and of comparable magnitude to those obtained in the full sample (Table 9). COVID-19 infection status remained non-significant (F(1, 202) = 0.006, *p* = 0.938), as did job rank, marital status, clinical unit, number of children, education, and sex. Restricting the sample to nurses who were demonstrably in practice during the peak pandemic period therefore did not alter any substantive conclusion of the study.

## 4. Discussion

The findings below are interpreted in light of the study’s observational, cross-sectional design. Because work stress and social support were reported retrospectively with reference to the pandemic period while professional commitment was reported as a current attitude, and because all measures were self-reported by the same respondents at a single time point, the associations described here indicate patterns of covariation rather than causal relationships. They are accordingly presented as provisional interpretations to be weighed against the limitations set out in Section 4.7.

### 4.1. COVID-19 Infection, Long COVID, and Professional Commitment

Hypothesis H1, which predicted that nurses who had contracted COVID-19 or reported long COVID symptoms would show lower professional commitment, was not supported: neither COVID-19 infection status nor the presence of long COVID symptoms was significantly associated with work stress, social support, or professional commitment scores. One interpretation is that nurses’ baseline professional investment and the social support they received from family and colleagues buffered any potential negative effect of infection on their professional commitment, allowing them to continue engaging in patient care despite the personal risk and experience of illness. This pattern suggests that nurses’ professionalism and social support networks may, to some extent, have offset the potential impact of COVID-19 infection risk on their clinical engagement.

### 4.2. Sociodemographic Factors, Work Stress, and Social Support in Relation to Professional Commitment

The second set of hypotheses received mixed support. As predicted by H2b, higher social support was associated with higher professional commitment, and, consistent with H2c, older age was associated with higher commitment. By contrast, H2a was not supported in the predicted direction: work stress was associated with professional commitment positively rather than negatively. The positive association between social support and commitment is consistent with previous findings that family and organizational support are linked to greater professional commitment and to greater certainty about remaining in the profession [9,11,12,13]. Similarly, the association between older age and higher commitment is consistent with earlier studies reporting that older nurses tend to show greater professional commitment [11,13]. The positive association between work stress and commitment, although unexpected relative to the most prior literature, may again reflect the unusual circumstances of the pandemic: nurses with strong professional investment and good social support may have been more willing to take on COVID-19-related responsibilities—and therefore report higher stress—while remaining, or even becoming, more committed to the profession as a result. This interpretation should nonetheless be advanced with considerable caution. The bivariate association between work stress and professional commitment was weak (r = 0.116), accounting for approximately 1.3% of the variance in commitment scores, and its statistical significance is attributable in large part to the size of the sample rather than to the magnitude of the effect. The finding is therefore best regarded as a small, statistically detectable departure from the negative association reported in most prior work, rather than as evidence of a substantively important positive relationship, and it requires replication before any confident theoretical or practical conclusions are drawn from it.

Taken together, these cross-sectional findings are consistent with the possibility that social support, age, and work stress are related to nurses’ professional commitment, although the observational design does not permit conclusions about the direction of these relationships; they tentatively suggest that social support in particular may help nurses sustain good stress tolerance—and, in turn, their professional commitment—during infectious disease outbreaks.

### 4.3. Comparison with the Prior Literature

The present findings both extend and diverge from prior empirical work on nurses’ professional commitment during the COVID-19 pandemic. Consistent with Duran et al. [13], who reported that pandemic-related factors were associated with nurses’ professional commitment in a Turkish sample, the present study similarly found a significant association between work stress and commitment. Notably, however, the direction of this association differed: Duran et al. reported associations consistent with the more commonly observed health-impairment pathway of the JD-R model, whereas the present study found a positive association, which we interpret (Section 4.5) as reflecting the motivational, resource-buffering pathway of the JD-R model instead. This divergence suggests that the relationship between work stress and professional commitment during large-scale infectious disease outbreaks may be more context-dependent than previously assumed, and may hinge on the relative strength of concurrent job resources such as social support.

The present study’s finding that social support was the strongest correlate of professional commitment aligns with Yu et al. [12], who reported that social support and resilience were positively associated with professional commitment and intention to stay among newly graduated nurses, and with Lu, Chang, and Wu [11], who found that social support and lower work stress were both associated with greater professional commitment among Taiwanese public health nurses prior to the pandemic. Taken together with the present findings, this convergence across pre-pandemic and pandemic-era samples suggests that social support functions as a comparatively stable resource for professional commitment across occupational contexts and time periods, consistent with the JD-R model’s proposition that resources exert a broadly protective, motivational effect regardless of the specific demands present. This interpretation is further reinforced by recent post-pandemic evidence: a 2024 meta-analysis of 38 studies (63,989 clinical nurses) confirmed a medium-sized protective association between social support and turnover intention [10], indicating that the resource role of social support documented here remains salient in the contemporary, post-acute-pandemic nursing workforce.

By contrast, the null association between COVID-19 infection status and professional commitment observed here differs from findings reported in nursing-student samples by Zhao et al. [6] and Tang et al. [8], both of whom found that pandemic-related experiences were associated with shifts in professional identity or commitment during this period. This divergence may reflect a substantive difference between nursing students, who are still in the process of forming their professional identity, and practicing clinical nurses, whose professional commitment—as reflected in the comparatively high descriptive scores observed in this sample (Table 4)—may already be well established and therefore more resistant to disruption by a single occupational exposure such as infection status.

### 4.4. Patterns of Scale Scores

On the work stress scale, PPE-related discomfort and infection-control difficulty/anxiety received the highest subscale scores, while fear of social isolation scored lowest (item-level rankings are given in Table S1). This pattern suggests that the physical burden and complexity of protective procedures—rather than social isolation per se—were the most salient stressors for these nurses, possibly reflecting a strong sense of unit cohesion and shared professional commitment that buffered concerns about social isolation.

On the social support scale, support from family/friends and colleagues was rated more highly than support from supervisors (Table S2), suggesting that nurses relied primarily on close personal relationships for psychological grounding during this high-stress period while perceiving relatively less support from administrative leadership. This gap may reflect a mismatch between the kind of support frontline staff most wanted (e.g., emotional reassurance) and the kind of support supervisors most readily provided (e.g., workload-reduction measures).

On the professional commitment scale, the professional-investment items scored highest and the retention items scored lowest (Table S3), indicating that nurses remained strongly engaged in providing high-quality care and pursuing continuing professional development, even though their long-term willingness to remain in the profession was comparatively more fragile. This fragility is plausibly related to a mismatch between compensation and workload that short-term pandemic hazard-pay measures may not have been sufficient to resolve.

### 4.5. Reliability of the Instruments and Inter-Scale Correlations

All three instruments showed good internal consistency in this sample (Table 2), consistent with or exceeding the reliability reported in their original validation studies [16,17,18]. Social support and professional commitment were positively correlated, in line with previous reports that family, peer, and organizational support are associated with greater professional commitment among nurses [9,11,12,13]. Work stress was also positively, though weakly, correlated with professional commitment—a pattern that runs counter to much of the prior literature, in which higher stress is more commonly associated with lower commitment. We speculate that, given the unprecedented nature of the COVID-19 outbreak, nurses who remained in clinical roles during this period may have done so out of a strong sense of professional mission, willingness to face the challenge, and close teamwork in confronting the outbreak together—factors that may have strengthened, rather than eroded, their professional commitment even as stress levels rose. This pattern can be interpreted within the JD-R framework as evidence that, in this sample, the motivational process associated with job resources (social support, professional investment) outweighed the health-impairment process typically associated with job demands (work stress), such that resources appear to have buffered rather than been overwhelmed by demands during the acute phase of the pandemic.

### 4.6. Strengths and Limitations of the Electronic Survey Method

Electronic data collection offered several practical advantages for this study: it was cost-effective (no printing, mailing, or manual data entry), convenient for shift-working nurses to complete at a time and location of their choosing, anonymous (which may have improved the honesty of responses), and amenable to automated data compilation, reducing transcription error. At the same time, an online format may have excluded staff with limited access to, or comfort with, electronic devices, introducing potential sampling bias. The anonymity of online responses, moreover, cannot fully rule out incomplete, careless, or inaccurate answers, and response rates to electronic surveys are often lower than for paper-based administration, which can complicate efforts to reach a target sample size.

### 4.7. Limitations

This study has several limitations. First, the sample was drawn from a single medical center in eastern Taiwan, which may limit the representativeness and generalizability of the findings to nurses in other regions or types of healthcare facilities. Second, the cross-sectional design cannot establish causal relationships or capture how professional commitment may have changed over time; nurses’ professional commitment after contracting COVID-19 may continue to evolve. Third, data collection took place after pandemic restrictions had eased, and participants were asked to report their work stress and social support with reference to the pandemic period (Section 2.4). These retrospective judgements are susceptible to recall bias: the intensity of remembered stress may have attenuated or, conversely, been amplified by subsequent reflection, and participants’ present attitudes toward the profession may have coloured their recollection of that period. Because professional commitment was measured as a current attitude while the two predictors were measured retrospectively, the observed associations should be interpreted as associations between remembered pandemic experience and present commitment rather than as contemporaneous relationships; prospective or repeated-measures designs would be required to establish the latter. Fourth, the eligibility criterion of ≥3 months of clinical tenure—set on clinical grounds, since newly hired nurses in Taiwan require approximately three months to complete probation and practise independently—permitted the inclusion of nurses who entered practice after the peak of the pandemic and whose retrospective reports therefore rest on limited direct exposure. The sensitivity analysis reported in Section 3.8, restricted to nurses with ≥3 years of tenure, produced results consistent with those of the full sample, indicating that this did not materially affect the findings. Fifth, the survey response rate was 39.8% (364/914); because participation was voluntary, nurses who chose to respond may have differed systematically from non-responders—for instance in professional commitment, workload, or the salience of their pandemic experience—and the resulting self-selection bias may limit the representativeness of the sample even within the study hospital. Sixth, the sociodemographic post hoc comparisons in Table 7 used least-significant-difference tests without correction for multiple comparisons, increasing the risk of Type I error; these comparisons should be regarded as hypothesis-generating, and confirmatory studies should apply a family-wise error correction (e.g., Bonferroni or Tukey) when testing the specific pairwise differences identified here. Seventh, although data collection occurred in 2023–2024, during a period when acute pandemic restrictions in Taiwan had already eased, this timing itself constitutes part of the study’s contribution: the sample captures nurses’ retrospective and ongoing professional commitment after having lived through the acute phase of the COVID-19 outbreak, a transitional period that is unlikely to be replicated and that offers a historically specific window not available to studies conducted either during the earliest, most acute phase of the pandemic or in the post-pandemic period once COVID-19 had been reclassified as an endemic disease in Taiwan. As such, the findings should be read as characterizing nurses’ professional commitment specifically during this transitional phase, rather than as a general, time-invariant account of pandemic-related occupational stress. Eighth, the approximately five-month data collection window (21 September 2023 to 28 February 2024) may have introduced temporal heterogeneity, as nurses’ work stress and social support could plausibly have fluctuated with concurrent shifts in hospital staffing levels, seasonal patient volume, or evolving institutional COVID-19 policies over this period. The cross-sectional design did not capture the specific month in which each participant responded, precluding a formal test of temporal trends within the sample. Future studies using a shorter data collection window, or explicitly modelling time of response as a covariate, would help clarify whether the associations reported here are stable across the data collection period or are sensitive to its timing. Ninth, the social support scale used in this study derives from an unpublished master’s dissertation, and no peer-reviewed publication reporting its psychometric validation could be identified; although the scale demonstrated excellent internal consistency in the present sample (Cronbach’s α = 0.970, Table 2), reliability alone does not establish construct validity, and the associations involving social support reported here should therefore be interpreted with corresponding caution and re-examined in future studies using instruments with published validity evidence. Finally, the distributional assumptions underlying the parametric analyses (normality and homogeneity of variance) were not formally tested; although the relatively large sample (*N* = 364) affords some robustness to moderate departures from normality for the *t*-tests, ANOVA, and regression analyses reported here, the absence of formal assumption checks is acknowledged as a limitation, and future analyses should verify these assumptions or employ robust or non-parametric alternatives. Finally, although Harman’s single-factor test suggested that common method variance was not a dominant influence in these data (Section 3.1), all measures were self-reported by the same respondents at a single time point; Harman’s test is a relatively insensitive diagnostic and cannot wholly exclude common method bias, so the observed associations may still be inflated to some degree relative to those that would be obtained using multi-source or multi-time-point designs.

## 5. Conclusions

This study examined whether contracting COVID-19 pneumonia affected clinical nurses’ professional commitment, using three scales measuring work stress, social support, and professional commitment, two of which (the work stress and professional commitment scales) have published psychometric validation, while the social support scale derives from an unpublished dissertation and is supported primarily by the internal-consistency evidence obtained in this sample. Professional commitment did not differ significantly by COVID-19 infection status, and neither did it differ by the presence of long COVID symptoms—a finding that runs counter to our initial hypothesis. Nurses in this sample reported relatively high professional commitment overall. Further analysis found that work stress and social support were both statistically associated with professional commitment, and that commitment differed by age group. These conclusions are offered with substantial caution. The cross-sectional design precludes any inference about causal direction; work stress and social support were reported retrospectively while commitment was reported as a current attitude; the response rate was 39.8%, so self-selection cannot be excluded; and the association between work stress and commitment, although statistically significant, was weak in magnitude. The present results should therefore be read as preliminary and exploratory, indicating associations that warrant confirmation in prospective, multi-site research rather than establishing findings on which practice or policy decisions should presently be based. Subject to such confirmation, they may serve as a starting point for hospital administrators considering how nurses’ professional commitment and workforce retention might be supported.

### Recommendations for Future Research and Practice

Future studies could consider more objective measures (e.g., physiological stress indicators) to reduce reliance on self-report and its associated recall bias; longitudinal or experimental designs to clarify causal relationships between infection, stress, social support, and professional commitment over time; and broader, multi-site samples spanning different regions and types of healthcare institutions to improve generalizability.

From a practice perspective, and acknowledging that the present cross-sectional evidence is preliminary and cannot establish that intervening on social support would change professional commitment, the association observed here may nonetheless offer a tentative basis for institutions considering how to strengthen psychological and organizational support for nursing staff—for example through counseling services, peer-support groups, improved workflow design, and adequate staffing and resources—to help nurses manage work stress more effectively. Institutions should also strengthen infection-prevention measures for frontline staff, including adequate personal protective equipment, regular health screening, vaccination access, and robust infection-control protocols, and should continue to monitor nurses’ professional commitment over time—for example through periodic job-satisfaction surveys, structured feedback channels, and career-development opportunities—so that support can be tailored to individual and unit-level needs. Whether such measures would in fact strengthen professional commitment remains an open question that the present design cannot answer, and one that future intervention or longitudinal studies would be needed to address.

## Figures and Tables

**Table 1:** Summary of measurement instruments.

Scoring and Original Reliability	Items	Dimensions	Instrument
5-point Likert (1 = strongly disagree to 5 = strongly agree); total score range 32–160, higher = greater stress. Subscale α = 0.84–0.90; overall α > 0.92.	32	Fear of social isolation; PPE-related discomfort; infection-control difficulty/anxiety; burden of patient care	Stress Scale for Healthcare Workers Caring for Patients with High-Risk Infectious Diseases [16]
6-point scale (0 = no such relationship to 5 = a great deal); total score range 0–105, higher = greater support. α = 0.78; test–retest r = 0.89.	21	Family/friends; colleagues/peers; supervisors (emotional, affirmational, instrumental support)	Social Support Scale for clinical nurses [17]
5-point Likert (1 = strongly disagree to 5 = strongly agree); total score range 19–95, higher = greater commitment. α = 0.91.	19	Professional identity; professional investment; professional retention	Hospital Nurses’ Job Satisfaction and Professional Commitment Scale [18]

Note: PPE = personal protective equipment. α = Cronbach’s alpha (original-study values).

**Table 2:** Internal consistency (Cronbach’s alpha) of the study instruments.

This-Study α	Original-Study α	Instrument
0.937	0.92	Stress Scale for Healthcare Workers Caring for Patients with High-Risk Infectious Diseases
0.970	0.89	Social Support Scale
0.941	0.91	Hospital Nurses’ Job Satisfaction and Professional Commitment Scale

Note: α = Cronbach’s alpha.

**Table 3:** Sociodemographic characteristics of participants (*N* = 364).

%	*n*	Category	Variable
9.1	33	Male	Sex
90.9	331	Female	
27.7	101	18–25	Age (years)
27.2	99	26–30	
14.8	54	31–35	
7.4	27	36–40	
12.1	44	41–45	
6.0	22	46–50	
4.7	17	≥51	
36.8	134	Married/previously married	Marital status
63.2	230	Never married	
68.1	248	None	Number of children
7.2	27	1	
18.4	67	2	
6.0	22	≥3	
15.4	56	Associate/college degree	Education
75.0	273	Bachelor’s degree	
9.6	35	Graduate degree or above	
3.8	14	Outpatient department	Clinical unit
45.6	166	General ward	
11.3	41	Emergency department	
16.7	61	Critical care unit	
22.6	82	Other	
3.3	12	<1	Years of service
18.1	66	1–2	
17.6	64	3–5	
22.0	80	6–10	
39.0	142	≥10	
29.4	107	N/N1	Job rank
31.9	116	N2	
15.1	55	N3	
4.9	18	N4	
4.4	16	HN/AHN	
14.3	52	NP	
86.8	316	Yes	COVID-19 infection
13.2	48	No	
45.9	145	Yes	Long COVID symptoms (*n* = 316)
54.1	171	No	

Note: N/N1, N2, N3, N4, HN, AHN, and NP denote the hospital’s internal clinical-ladder and nurse-practitioner job-rank categories (ascending seniority from N/N1 to HN/AHN; NP = nurse practitioner).

**Table 4:** Descriptive statistics for scale and subscale scores.

Item SD	Item Mean	Total SD	Total Mean	Max	Min	Items	Scale/Subscale
0.7	3.8	21.6	120.3	160	32	32	Work stress scale (total)
0.7	4.3	5.8	34.5	40	8	8	PPE-related discomfort
0.8	4.0	5.3	27.9	35	7	7	Burden of patient care
1.0	3.4	9.6	34.4	50	10	10	Fear of social isolation
0.8	3.4	5.9	23.5	35	7	7	Infection-control difficulty/anxiety
1.0	3.1	20.8	65.1	105	0	21	Social support scale (total)
1.1	3.6	8.0	25.0	35	0	7	Family/friends
1.1	3.1	7.7	21.4	35	0	7	Colleagues/peers
1.1	2.6	8.0	18.7	35	0	7	Supervisors
0.7	3.8	13.4	72.6	95	19	19	Professional commitment scale (total)
0.7	4.4	3.4	21.9	25	5	5	Participation/engagement
0.7	4.2	2.8	16.8	20	4	4	Contribution
0.9	3.5	2.8	10.4	15	3	3	Compliance
1.0	3.4	3.9	13.6	20	4	4	Cognition/awareness
1.1	3.3	3.2	10.0	15	3	3	Retention

Note: PPE = personal protective equipment. SD = standard deviation. The professional-commitment subscale labels (participation/engagement, contribution, compliance, cognition/awareness, retention) reflect the five item clusters of the original instrument and map onto its three broader dimensions (professional identity, investment, and retention) described in Section 2.3.4.

**Table 5:** Independent-sample *t*-test results for COVID-19 infection status and long COVID symptoms.

*p*	t	SD	Mean	*n*	Group	Scale
0.478	0.710	21.4	120.0	316	COVID-19 infected	Work stress
		23.0	122.4	48	Not infected	
0.177	1.353	20.9	64.5	316	COVID-19 infected	Social support
		20.0	68.9	48	Not infected	
0.619	0.498	13.5	72.5	316	COVID-19 infected	Professional commitment
		12.8	73.5	48	Not infected	
0.112	−1.594	19.8	122.1	145	Long COVID symptoms (*n* = 316)	Work stress
		22.6	118.2	171	No long COVID symptoms	
0.637	0.472	20.6	63.9	145	Long COVID symptoms	Social support
		21.2	65.1	171	No long COVID symptoms	
0.397	0.848	14.4	71.8	145	Long COVID symptoms	Professional commitment
		12.6	73.1	171	No long COVID symptoms	

Note: SD = standard deviation. The long-COVID comparison was restricted to the 316 participants who reported COVID-19 infection.

**Table 6:** Pearson correlation matrix among the three scales.

3. Professional Commitment	2. Social Support	1. Work Stress	Variable
		1.000	1. Work stress
	1.000	−0.006 [0.903]	2. Social support
1.000	0.261 [<0.001] ***	0.116 [0.027] *	3. Professional commitment

Note: Cell values are Pearson’s r [*p*-value]. * *p* < 0.05; *** *p* < 0.001.

**Table 7:** One-way ANOVA of sociodemographic variables and professional commitment.

Omnibus F (*p*)	*p*	SE	Mean Diff.	Comparison (vs. Reference)	Variable
28.191 (<0.001)				Reference: 18–30	Age (years)
	<0.001	1.6	−6.5	31–40	
	<0.001	1.6	−11.8	≥41	
5.810 (<0.001)				Reference: HN/AHN	Job rank
	<0.001	3.5	13.0	N/N1	
	0.001	3.4	11.0	N2	
	0.007	3.7	10.0	N3	
	0.729	4.4	1.5	N4	
	0.119	3.7	5.8	NP	
15.766 (<0.001)				Reference: ≥10 years	Years of service
	<0.001	1.8	10.7	0–2 years	
	<0.001	1.9	7.6	3–5 years	
	<0.001	1.8	8.7	6–10 years	
8.588 (<0.001)				Reference: graduate degree	Education
	<0.001	2.3	9.7	Bachelor’s degree	
	0.003	2.8	8.3	Associate/college degree	
1.173 (0.323)				Reference: general ward	Clinical unit
	0.223	3.7	−4.5	Outpatient department	
	0.125	2.3	−3.6	Emergency department	
	0.748	2.0	−0.6	Critical care unit	
	0.130	1.8	−2.7	Other	

Note: Mean differences (Reference − Comparison group) and LSD post hoc *p*-values are from the one-way ANOVA reported in the source analysis. Negative mean differences indicate that the comparison group scored higher than the reference group. The omnibus F-value for “Job rank” (F(5, 358) = 5.810, *p* < 0.001) was re-run to correct a duplication artifact in the original source tables, in which this value had been erroneously copied from the “Age” ANOVA output; the pairwise post hoc comparisons for Job rank reported above are unaffected by this correction.

**Table 8:** Multiple regression model: significance of predictors entered (Type III sum-of-squares ANOVA).

Significant?	*p*	F	df	Predictor
Yes	<0.001	6.550	22	Corrected model
—	<0.001	135.127	1	Intercept
Yes	<0.001	42.677	1	Social support
Yes	0.006	7.620	1	Work stress
Yes	0.002	6.385	2	Age
No	0.266	1.293	5	Job rank
No	0.214	1.502	3	Years of service
No	0.706	0.142	1	Marital status
No	0.059	2.297	4	Clinical unit
No	0.620	0.247	1	Number of children
No	0.526	0.402	1	COVID-19 infection status
No	0.212	1.561	1	Sex
No	0.228	1.483	2	Education

Note: R^2^ = 0.297 (adjusted R^2^ = 0.252); error df = 341. “Long COVID symptoms” was dropped from this model because it was collinear with, and effectively redundant with, COVID-19 infection status; with this variable removed, the model was no longer rank-deficient and all predictors—including COVID-19 infection status—were estimable. The near-identical F-values and *p*-values for work stress, social support, and the sociodemographic predictors relative to the originally reported (rank-deficient) model indicate that this correction did not materially change the substantive conclusions.

**Table 9:** Final regression coefficients for predictors significantly associated with professional commitment.

95% CI	*p*	t	SE	B	Predictor
[48.307, 70.473]	<0.001	10.541	5.634	59.390	Intercept
[0.144, 0.268]	<0.001	6.533	0.032	0.206	Social support
[0.023, 0.135]	0.006	2.761	0.029	0.079	Work stress
[−17.225, −4.338]	0.001	−3.291	3.276	−10.781	Age: 18–30 years (ref. ≥ 41 years)
[−9.675, −1.837]	0.004	−2.889	1.993	−5.756	Age: 31–40 years (ref. ≥ 41 years)

Note: B = unstandardized regression coefficient; SE = standard error; CI = confidence interval. Coefficients are from the corrected model (Section 3.7) in which long COVID symptoms were excluded due to collinearity with COVID-19 infection status. All other predictors entered in Table 8 (job rank, years of service, marital status, clinical unit, number of children, COVID-19 infection status, sex, and education) were not significantly associated with professional commitment and are omitted from this table; full parameter estimates for all predictors are available from the corresponding author on request.

## Data Availability

The data presented in this study are available upon request from the corresponding author due to privacy and ethical restrictions associated with the data-sharing terms approved by the Research Ethics Committee. The original-language study questionnaire and scale-use permission letters are available from the corresponding author upon request.

## Supplementary Materials

The following supporting information can be downloaded at: https://www.mdpi.com/article/10.3390/nursrep16090309/s1, Table S1. Item ranking, COVID-19 Pandemic-Related Work Stress Scale (32 items, N = 364); Table S2. Item mean scores by subscale, Social Support Scale (21 items, N = 364); Table S3. Item ranking, Professional Commitment Scale (19 items, N = 364); Table S4: STROBE Statement—Checklist of items that should be included in reports of cross-sectional studies.

## Public Involvement Statement

There was no public involvement in any aspect of this research.

## Guidelines and Standards Statement

This study is reported in accordance with the Strengthening the Reporting of Observational Studies in Epidemiology (STROBE) guidelines for cross-sectional studies; a completed STROBE checklist is provided as Supplementary Materials (Table S4).

## Use of Artificial Intelligence

During the preparation of this manuscript, the authors used Claude (Anthropic, San Francisco, CA, USA) to assist with translating into English the Chinese-language content of the first author’s own unpublished master’s thesis, on which this article is based, and with drafting and reformatting the text to conform to the journal’s structural and reference requirements. Successive model versions were used over the preparation period, most recently Claude Opus 5. The authors have reviewed and edited the output and take full responsibility for the content of this publication.

## References

[1] Chowdhury S.D., Oommen A.M. (2020). Epidemiology of COVID-19. J. Dig. Endosc..

[2] Gholami M., Fawad I., Shadan S., Rowaiee R., Ghanem H., Khamis A.H., Ho S.B. (2023). The COVID-19 pandemic and health and care workers: Findings from a systematic review and meta-analysis (2020–2021). Int. J. Public Health.

[3] Taiwan Centers for Disease Control, Ministry of Health and Welfare. Disease Information: COVID-19. https://www.cdc.gov.tw/Category/Page/vleOMKqwuEbIMgqaTeXG8A.

[4] Falatah R. (2021). The impact of the coronavirus disease (COVID-19) pandemic on nurses’ turnover intention: An integrative review. Nurs. Rep..

[5] Varghese A., George G., Kondaguli S.V., Naser A.Y., Khakha D.C., Chatterji R. (2021). Decline in the mental health of nurses across the globe during COVID-19: A systematic review and meta-analysis. J. Glob. Health.

[6] Zhao L., Su Y., Jiang N., Zhou F., Liao L., Liu Y. (2022). Changes in professional commitment of undergraduate nurse students before and after internship: A longitudinal study. BMC Med. Educ..

[7] Lu K.Y., Pan S.M., Lee L.L., Shia L.Y., Chang Y.Y. (2001). The influence of professional commitment on turnover intention. Kaohsiung J. Med. Sci..

[8] Tang M., Sun Y., Zhang K., Luo R., Liu Y., Sun H., Zhou F. (2022). Associated factors of professional identity among nursing undergraduates during COVID-19: A cross-sectional study. Int. J. Nurs. Sci..

[9] Duran S., Celik I., Ertugrul B., Ok S., Albayrak S. (2021). Factors affecting nurses’ professional commitment during the COVID-19 pandemic: A cross-sectional study. J. Nurs. Manag..

[10] Chen Y., Zhou X., Bai X., Liu B., Chen F., Chang L., Liu H. (2024). A systematic review and meta-analysis of the effectiveness of social support on turnover intention in clinical nurses. Front. Public Health.

[11] Lu K.Y., Chang L.C., Wu H.L. (2007). Relationships between professional commitment, job satisfaction, and work stress in public health nurses in Taiwan. J. Prof. Nurs..

[12] Yu H., Huang C., Chin Y., Shen Y., Chiang Y., Chang C., Lou J. (2021). The mediating effects of nursing professional commitment on the relationship between social support, resilience, and intention to stay among newly graduated male nurses: A cross-sectional questionnaire survey. Int. J. Environ. Res. Public Health.

[13] Chang H.Y., Shyu Y.I., Wong M.K., Friesner D., Chu T.L., Teng C.I. (2015). Which aspects of professional commitment can effectively retain nurses in the nursing profession?. J. Nurs. Scholarsh..

[14] Demerouti E., Nachreiner F., Bakker A.B., Schaufeli W.B. (2001). The job demands-resources model of burnout. J. Appl. Psychol..

[15] Bakker A.B., Demerouti E. (2017). Job demands-resources theory: Taking stock and looking forward. J. Occup. Health Psychol..

[16] Chuang P.Y., Luo M.F. (2005). Development and testing of a stress scale for healthcare workers caring for patients with high-risk infectious diseases: The case of SARS. Taiwan J. Public Health.

[17] Ni L.C. (1998). An Exploration of the Quality of Life and Related Factors for Medical-Surgical Nurses. Unpublished Master’s Thesis.

[18] Lin C.J., Wang H.C., Li T.C., Huang L.C. (2007). Reliability and validity of nurses’ job satisfaction scale and nurses’ professional commitment. Mid-Taiwan J. Med..

