# Supplementary material for: COVID-19 Infection and Professional Commitment Among Clinical Nurses: A Cross-Sectional Study of Work Stress and Social Support in Eastern Taiwan
Source: Nurs Rep. 2026 Aug 30;16(9):309. doi: 10.3390/nursrep16090309 (PMC13610304; doi:10.3390/nursrep16090309)
Supplement: Supplementary file 1 [file nursrep-16-00309-s001.zip › nursrep-4419101-supplementary.pdf]

Supplementary Materials

Table S1. Item ranking, COVID-19 Pandemic-Related Work Stress Scale (32 items, N = 364).

Item	Statement	M	SD
13	Wearing a face shield and hair cover impairs my vision.	4.5	0.9
12	Wearing protective equipment is hot, uncomfortable, and restricts movement.	4.5	0.8
4	I worry about infecting family or friends with COVID-19.	4.4	1
15	Using the restroom during work is inconvenient.	4.4	1
16	Eating and drinking during work are restricted.	4.4	0.9
18	Frequent handwashing or disinfectant use makes my hands rough and chapped.	4.3	1
17	I worry that wearing a mask will cause facial skin allergy or pressure injury.	4.2	1
30	I worry about patients who do not cooperate with treatment (e.g., self-removing tubes, trying to leave the ward).	4.2	1
14	Wearing protective equipment creates a communication barrier.	4.2	1
27	Limited staff allowed into isolation rooms means I cannot get sufficient assistance.	4.2	0.9
26	Putting on protective equipment is time-consuming and delays addressing patient needs.	4.2	1
11	Wearing an N95 or P100 mask makes breathing difficult.	4	1.2
5	I worry that, if infected, I would be separated from my children/family.	3.8	1.3
28	My current workload is excessive.	3.8	1.1
31	I worry about being unable to address patients' and families' psychological/emotional needs.	3.8	1.1
32	I am afraid of families' negative emotions resulting from isolation regulations and visitation bans.	3.8	1.1
29	I am afraid of patients' conditions worsening or of patient death.	3.8	1.1
19	I am afraid of becoming infected with COVID-19 myself.	3.8	1.2
1	I worry about developing symptoms suggestive of COVID-19 (cold-like symptoms, fever, diarrhea).	3.8	1.2
25	As a professional responsibility, caring for patients cannot be refused.	3.8	1.2
22	Infection-control measures and related information change frequently, and I cannot keep up.	3.7	1.1
6	I worry about being unable to care for my children/family, and about arranging their care.	3.7	1.4
3	I worry about restrictions on my social life and activities.	3.6	1.3
2	I worry about being placed under quarantine.	3.4	1.4

24	I worry that protective-equipment supplies are insufficient (e.g., shortages of masks or gowns).	3.3	1.4
21	I worry about whether existing protective measures are safe enough.	3.3	1.3
7	I worry that I or my family would be discriminated against or ostracized.	3.2	1.4
8	I worry about having nowhere suitable to stay after work.	3.2	1.4
20	My knowledge of COVID-19 is insufficient.	2.8	1.2
23	My technique for using protective measures is not proficient.	2.8	1.2
10	I am afraid to discuss my work in public or reveal my workplace and job duties.	2.8	1.4
9	Family/friends do not support my nursing work and ask me to refuse COVID-19 patients or resign.	2.5	1.4

Note: Items are ranked in descending order of mean score (1 = strongly disagree, 5 = strongly agree).

Table S2. Item mean scores by subscale, Social Support Scale (21 items, N = 364).

Support source	Item dimension	M	SD
Family and friends	Trust	3.8	1
Family and friends	Respect	3.7	1
Family and friends	Caring	3.7	1
Family and friends	Approval/affirmation	3.7	1
Family and friends	Emotional support	3.7	1
Family and friends	Short-term instrumental support	3.7	1.1
Family and friends	Long-term instrumental support	3.6	1.1
Colleagues/peers	Trust	3.4	1
Colleagues/peers	Respect	3.4	1
Colleagues/peers	Caring	3.4	1
Colleagues/peers	Approval/affirmation	3.3	1
Colleagues/peers	Emotional support	3.3	1
Colleagues/peers	Short-term instrumental support	3.1	1.1
Colleagues/peers	Long-term instrumental support	3	1.1
Administrative supervisors	Respect	3	1.1
Administrative supervisors	Caring	3	1.1
Administrative supervisors	Emotional support	3	1.1
Administrative supervisors	Trust	3	1
Administrative supervisors	Approval/affirmation	3	1
Administrative supervisors	Short-term instrumental support	2.8	1.1
Administrative supervisors	Long-term instrumental support	2.8	1.1

Note: Items within each support source are ranked in descending order of mean score (1 = none at all, 6 = a great deal).

Table S3. Item ranking, Professional Commitment Scale (19 items, N = 364).

Item	Statement	M	SD
8	Nursing is a professional occupation that requires professional training.	4.6	0.8
10	I work conscientiously in my nursing position.	4.4	0.8
11	I think about how to perform nursing work well.	4.4	0.8

14	I devote myself to caring for patients to help them recover.	4.3	0.8
15	I make every effort to improve patients' self-care ability.	4.3	0.8
9	I try to make full use of my abilities in nursing work.	4.3	0.8
12	I try to understand each patient's individual needs to develop a care plan.	4.2	0.8
16	I am willing to overcome difficulties encountered in my work.	4.2	0.8
13	I care for patients as if they were my own family or friends.	4	0.9
2	Being a nurse is meaningful to me.	3.9	1
7	Nursing work enriches my personal life experience.	3.7	1
6	Telling others that I am a nurse is something to be proud of.	3.6	1.1
17	Even without promotion opportunities, I would continue working as a nurse.	3.6	1.2
19	After marriage, I would continue working as a nurse.	3.5	1.2
4	I enjoy talking with others about nursing work.	3.4	1.1
1	Nursing is the work I am most interested in doing in my life.	3.4	1.2
5	Working as a nurse allows me to realize my ideals and aspirations.	3.1	1.2
3	I am willing to make nursing my lifelong career.	3	1.3
18	Even if somewhat dissatisfied with my salary, I would continue working as a nurse.	2.9	1.3

Note: Items are ranked in descending order of mean score (1 = strongly disagree, 5 = strongly agree).

Table S4. STROBE Statement—Checklist of items that should be included in reports of ***cross-sectional studies.***

	Item No	Recommendation	Page No
**Title and abstract**	1	(*a*) Indicate the study's design with a commonly used term in the title or the abstract	2
		(*b*) Provide in the abstract an informative and balanced summary of what was done and what was found	2
		**Introduction**	
Background/rationale	2	Explain the scientific background and rationale for the investigation being reported	3–5

Objectives	3	State specific objectives, including any prespecified hypotheses	5
**Methods**			
Study design	4	Present key elements of study design early in the paper	6
Setting	5	Describe the setting, locations, and relevant dates, including periods of recruitment, exposure, follow-up, and data collection	6–7
Participants	6	(*a*) Give the eligibility criteria, and the sources and methods of selection of participants	7
Variables	7	Clearly define all outcomes, exposures, predictors, potential confounders, and effect modifiers. Give diagnostic criteria, if applicable	7–9
Data sources/ measurement	8*	For each variable of interest, give sources of data and details of methods of assessment (measurement). Describe comparability of assessment methods if there is more than one group	7–9
Bias	9	Describe any efforts to address potential sources of bias	7, 10, 22–23
Study size	10	Explain how the study size was arrived at	6
Quantitative variables	11	Explain how quantitative variables were handled in the analyses. If applicable, describe which groupings were chosen and why	10
Statistical methods	12	(*a*) Describe all statistical methods, including those used to control for confounding	10
		(*b*) Describe any methods used to examine subgroups and interactions	10, 15, 18
		(*c*) Explain how missing data were addressed	7
		(*d*) If applicable, describe analytical methods taking account of sampling strategy	7
		(*e*) Describe any sensitivity analyses	10, 18
**Results**			
Participants	13*	(a) Report numbers of individuals at each stage of study—eg numbers potentially eligible, examined for eligibility, confirmed eligible, included in the study,	7, 12

		completing follow-up, and analysed	
		(b) Give reasons for non-participation at each stage	7
		(c) Consider use of a flow diagram	n/a
Descriptive data	14*	(a) Give characteristics of study participants (eg demographic, clinical, social) and information on exposures and potential confounders	12
		(b) Indicate number of participants with missing data for each variable of interest	7, 17
Outcome data	15*	Report numbers of outcome events or summary measures	14
Main results	16	(*a*) Give unadjusted estimates and, if applicable, confounder-adjusted estimates and their precision (eg, 95% confidence interval). Make clear which confounders were adjusted for and why they were included	16–17
		(*b*) Report category boundaries when continuous variables were categorized	10, 12
		(*c*) If relevant, consider translating estimates of relative risk into absolute risk for a meaningful time period	n/a
Other analyses	17	Report other analyses done—eg analyses of subgroups and interactions, and sensitivity analyses	14–18
**Discussion**			
Key results	18	Summarise key results with reference to study objectives	19–21
Limitations	19	Discuss limitations of the study, taking into account sources of potential bias or imprecision. Discuss both direction and magnitude of any potential bias	22–24
Interpretation	20	Give a cautious overall interpretation of results considering objectives, limitations, multiplicity of analyses, results from similar studies, and other relevant evidence	19–21, 24
Generalisability	21	Discuss the generalisability (external validity) of the study results	23
**Other information**			

Funding	22	Give the source of funding and the role of the funders for the present study and, if applicable, for the original study on which the present article is based	26

*Give information separately for exposed and unexposed groups.

Note: An Explanation and Elaboration article discusses each checklist item and gives methodological background and published examples of transparent reporting. The STROBE checklist is best used in conjunction with this article (freely available on the Web sites of PLoS Medicine at http://www.plosmedicine.org/, Annals of Internal Medicine at http://www.annals.org/, and Epidemiology at http://www.epidem.com/). Information on the STROBE Initiative is available at www.strobe-statement.org.
